# The role of open standards in catalysing knowledge transfer to deliver climate adaptive care

**DOI:** 10.1038/s41746-024-01401-4

**Published:** 2025-04-07

**Authors:** Maeghan Orton, Olivia Swann, Gabrielle Samuel, Peter Drury, Dimitrios Kalogeropoulos, David Pencheon, Kristina Celentano, Babajide Babayeju, Liz Grant

**Affiliations:** 1 Global Health Academy, Usher Institute, University of Edinburgh, Edinburgh, UK; 2 Centre for Medical Informatics, University of Edinburgh, Edinburgh, UK; 3 Department of Global Health and Social Medicine, King’s College London, London, UK; 4 Drury Consulting Ltd, York, United Kingdom; 5 Global Health & Digital Innovation Foundation, London, UK; 6 University College London, Global Business School for Health, London, UK; 7 University of Exeter, Exeter, United Kingdom; 8 Koralaide Consulting LLC, Washington, DC USA; 9 University of Ilorin, Illorin, Nigeria

**Keywords:** Governance, Developing world, Risk factors, Epidemiology

## Abstract

As climate change threatens to destroy health gains, digital health provides infrastructure that is well-placed to offer patient-centred health insights. These insights are important to advance research to explore the intersection of climate and health. We present a proposal to leverage open data standards to more seamlessly collect, exchange, and use a combination of environmental and health data to assess climate-health risks to improve patient and population outcomes.

## Introduction

Climate change is threatening many health gains of the last century, with adverse environmental conditions likely to result in an additional 12.5 million deaths annually, and predictions stating that up to four billion people globally will be at risk due to the direct and indirect climate impacts of 2050^1,2^. The burden of deaths, diseases, and the socioeconomic and healthcare costs of the climate crisis on lives, livelihoods and livestock, affect those in lower and middle-income countries (LMICs) most deeply, and those with the least resources are the most vulnerable^3^. The differential impacts of climate change on individual health outcomes based on their health history and socioeconomic context require a focused approach to understand and respond to climate-related morbidity and mortality.

At present, international agencies including the World Bank focus on population-level indicators to measure the effect of climate change on population groups and to develop climate-health models to predict, diagnose, and associate evidence to treatments^3^. While this approach is a first step to establish a climate-informed health policy, it fails to consider patient-centred contexts, or the potentially cumulative nature of climatic hazards on a specific individual’s health over time. This approach risks misrepresenting the complex adversities that may be faced by particular high-risk communities experiencing climate hazards, defined as droughts, heat waves, and other significant weather events. These risks include both increased and new infectious diseases, displacement due to climate-related weather events, and new or unforeseen complications to chronic ailments including diabetes and asthma^4–6^ There is a critical need to shift towards digital health solutions that collect patient-level data over time and across diseases, and align this data with key climate and environmental indicators^2,5^.

The degree to which climate events directly affect physical and/or mental health, or impact one or more pre-existing health conditions, requires a new approach to epidemiological analysis^7^. Essential to understanding and addressing the interaction of climate on patient health is the documenting and interpreting of patient-centric, timely, and comprehensive health data which can be linked to key climate and environmental variables. Once these key variables are agreed, a climate-informed approach to epidemiological analysis can then be mobilised through well-defined research to enable evidence ecosystems to develop^8,9^.

To provide such patient-centred data representative of specific at-risk communities, scaled and sensitive digital health infrastructures are needed to characterise associations between climate hazards and specific health conditions in relation to patient-level outcomes. With an ever-increasing array of equipment, digital platforms and applications within healthcare that allow the collection and analyses of vast amounts of data, digital health ecosystems now offer a key foundation on which to collect, organize and interpret patient data with the goal of building evidence-informed, resilient health care systems prepared to face the climate crisis^5,10–12^.

A challenge for health systems is identifying subpopulations and individual patients most susceptible to the negative impacts of climate change^6,12,13,14^, thus enabling the provision of specific and appropriate care to those most in need^10^. New and poorly understood risk factors are likely to further impact those who are already vulnerable to climate-related risk factors^1,15^, and the existing health data sets are not easily accessible or comparable to the research community tasked with analysis at the patient level within and between countries^16^. Environmental variables are not yet documented in an agreed format, therefore limiting the ability of researchers and policy makers to understand and interpret the intersection of human health and climatic hazards. Health data managers must agree on an approach to organise and link patient datasets generated from digital health tools to document and interpret health impacts attributable to climate change, permitting insights to shape climate-resilient health systems^4,9,11,17^. What is more, evidence gaps need to be understood to permit specific and high-value research to be funded, conducted and evaluated^1^. The health data infrastructure in both low- and middle-income countries (LMICs) and high-income countries (HICs) must be equipped to maximise these opportunities^10^.

We argue that a significant shift towards patient-level data, alongside an agreed ontology of health-focused climatic and environmental data, is required. With access to well-labelled individual-level data, more accurate assessments of risks and impacts of climate on individual patient health can be understood. The SMART (Standards-based, Machine-readable, Adaptive, Requirements based and Testable) Guidelines are part of a broader data and technology approach of open standards, open technologies, open architecture, and open content, referred to as a Full-STAC (Standards, Technologies, Architectures and Content) framework^10,16^. This framework provides an opportunity to uniformly collect, share and use/re-use patient-level data in a common framework to understand the interactions of individual -level health and environmental and climatic hazards.

## The use of Open Standards to speed up evidence cycles for clinical guidance

The WHO develops global guidelines for healthcare delivery to ensure evidence regarding best-practices in clinical medicine is appropriately incorporated into healthcare delivery for both LMICs and HICs. Guidance is organised into intervention areas, including climate-focused topics like heat health adaptation plans^3^, and updated roughly every five years to ensure that the clinical standards of care reflect advances in medical & scientific understanding. Once guidelines are updated, country-level committees are tasked with localising this guidance to national contexts, and formalising into health policy. This can take between two and ten years. These localised guidelines become the framework for healthcare delivery for each country, allowing current best practices to be incorporated into routine clinical programs for individuals, and represent at minimum seven years of cumulative effort^13^.

The time-intensive nature of the guideline process demonstrates a significant population level risk due to the rapid advancements of climate change and environmental degradation^1,18^, and the dynamic interaction between human health and the environment which requires continuous monitoring^5,17,19,20^. As an example, essential guidance like heat-health was last updated in 2008^21^. Since last updated, incidents of extreme heat have become daily events, with new and numerous heat health conditions impacting mortality and morbidity^5^, Half of the world’s population and approximately one billion workers are at risk due to these extreme heat events, and individual risk profiles are compounded due to ageing, increased urbanisation and changes in socioeconomic status. During pregnancy, heat-related risks are specific to a pregnant person’s gestational stage, and require close monitoring during extreme heat events. Significantly, within tropical climates, increased warming means there is a risk of reaching the physiological limits of heat tolerance in a specific location^5,15,21^.

**Fig. 1:**
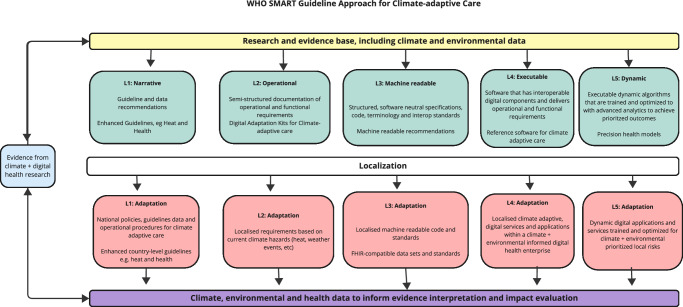
WHO Standards-based, Machine-readable, Adaptive, Requirements-based and Testable Guidelines Approach for Climate-adaptive Care. Evidence of the influence of climate and environment on health outcomes is health impacts (blue) generated by research generated from digital health platforms (yellow); WHO Guidelines are produced that follow climate-related clinical practice recommendations (green); localized guidelines are developed considering direct health impacts mediated by primary environmental impacts of climate change (red); and climate, environmental and health data resulting from digital health programs built on SMART guidelines inform further guideline development processes (purple); tertiary effects (upward-pointing arrows). Adapted from {Mehl, 2021} under Creative Commons V.4 licensing from Mehl G, et al.^26^.

Standard-based health data can help to improve both the cost effectiveness and efficiency of digital health^10,11,22^, One of the latest emerging standards for the exchange of health data is the standard Fast Healthcare Interoperability Resources^18,23^ (FHIR). The SMART Guidelines, recently endorsed by the WHO as a unifying resource for digital health application development, are built on the FHIR standard, and allow software developers to access digitised guideline-based content for use in digital health application development, clinical outcome research and climate and environmental monitoring via digital health tools^10^. Digital health tools, when built on standards like FHIR, provide necessary and critical health information infrastructure to advance analysis of patient impacts from population to patient level (See – Fig. 1.)

## WHO SMART Guidelines approach represents a national guideline development process built on a foundation of data and operational insights

**Fig. 2:**
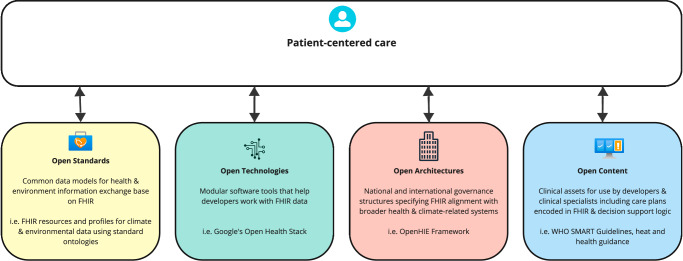
Climate-informed patient-centred care. Taken from {Mehl, 2023} with written permission from author and adapted under Creative Commons License V.4. Patient-centred care is enabled for climate-adaptive care as a result of climate and environmental data enhancing Open Standards (yellow); efficiencies gained by modularizing software to allow developers to work with FHIR data (green); national architectures which provide specific and clear governance models for both health and climate data use (red); content including resulting care plans which can be transparently shared, evaluated and understood (blue). Adapted under Creative Commons license 4.0 from Mehl et al. A full-STAC remedy for global digital health transformation: open standards, technologies, architectures, and content. *Oxford Open Digital Health*. 1, 1–5 (2023).

We suggest that SMART guidelines, when built on patient-centred data stored in an FHIR-compatible format (See Fig. 2,) can help organise and establish specific and sensitive data sets to understand climate-health interactions for use in both research and health policy development. A key benefit of FHIR-based datasets is the ability to document, agree and align on standard measures and terminology used in data collection and analysis^9,24^. When deployed either as a stand-alone deployment or at scale, this approach can be used to establish patient-centred care (See Fig. 2.) In addition, the SMART guidelines infrastructure is flexible enough due to the dynamic nature of FHIR as a standard to establish shared data concepts for use in climate - health modelling scenarios which document the specific contexts and conditions of climate-impacted communities and individuals in both LMIC and HIC settings^23^.

To support health care delivery to anticipate and respond to climate-affected health conditions, there is an immediate need for patient-specific evidence to inform clinical care provision and support health policy development. For these climate and environmentally sensitive guidelines, there is a need for a complimentary initiative that has its own benefits and risks to improve the specificity and sensitivity of these guidelines. For LMIC countries, the cost of developing fully scaled digital health programs can be prohibitive, although there are scaled implementations focused on highly vulnerable groups like pregnant persons and children under five available which can be included^9^. As a cost-saving measure, both low- and high-income countries would benefit from focusing on available data sets from highly vulnerable groups as a focused first step.

To facilitate this transition, open-source resources including FHIR PIT (Health Level 7 Fast Healthcare Interoperability Resources Patient data Integration Tool) have been developed to enable the evaluation of environmental exposure on individual patients^24^. At present, scaled evaluations are limited. FHIR PIT has been used to evaluate the impact of climate-related changes in air quality on asthmatic patient health in the United States. Initial evaluations were able to demonstrate the ability to evaluate large sets of anonymised patient data (n – 160,000), integrating patient-level health data with environmental data to expose and assess the impact of airborne particulate matter on patient health. Importantly, the evaluation surfaced vulnerabilities based on racial disparities in asthma exacerbation, exposing race as a patient-level variable to explore further in additional climate and health research.

### Recommendations and next steps: Establishing shared and dynamic climate health infostructure

The introduction of environmental exposure to patient-level data in an FHIR format is a compelling proof of concept to pave the way for the SMART Guidelines infrastructure to include climate and environmental exposure data as a resource for FHIR-based digital health platforms. As a next step, a Delphi study should be conducted to help reach a consensus in the field about specific environmental and climate variables to support the development of a formal ontology to inform further categorization of concepts and relationships at the intersection of climate and health^25^. If introduced to the FHIR PIT repository, this approach could enable continuous and scaled evaluation of the intersection of human health and climate hazards.

It is vital that such a study include stakeholders with expertise in environmental exposures, infectious disease, and SMART guideline development to ensure the timely inclusion of findings into the WHO Guideline process^26^. A preliminary list of environmental and climate variables could include (1) excess heat^5^, (2) drought in locations where drought was less severe^1,3^ (3) presence of wildfires^1,18^ (4) flooding where rain falls on already saturated land^1,19^ (5) rises in sea levels^1,6,18^ and (6) changes in ocean climate, both excessive heat and excessive cold^2^. For each of these variables, environmental and climate data sets would need to be evaluated to as part of the Delphi study protocol to determine localised ranges based on location and historic data^1,2,25,22,24^ An evaluation should also include an assessment of the availability of these data sets in LMIC and HIC contexts. For these data sets to be of use in digital health deployments, a final recommendation of this comment is to agree on the inclusion of GIS and location data for patient-level data in a minimum FHIR patient resource, collected over time. Patient-level data that is linked to climate and environmental indicators require location as a minimum data point for comparison, and this data element, is a first step to deliver research-informed and timely clinical care guidelines^1,25^^,^^27^.

To mitigate the impact of the climate events on patient health, health policy makers and practitioners and patients themselves require actionable guidance informed by multi-dimensional data that extends beyond national boundaries. Digital health platforms are a foundation for generating and interpreting this data to deliver quality, climate-adaptive care care.
